# Predicting critical state after COVID-19 diagnosis: model development using a large US electronic health record dataset

**DOI:** 10.1038/s41746-021-00482-9

**Published:** 2021-07-20

**Authors:** Mike D. Rinderknecht, Yannick Klopfenstein

**Affiliations:** IBM Switzerland Ltd, Zurich, Switzerland

**Keywords:** Viral infection, Risk factors, Prognosis, Computational science

## Abstract

As the COVID-19 pandemic is challenging healthcare systems worldwide, early identification of patients with a high risk of complication is crucial. We present a prognostic model predicting critical state within 28 days following COVID-19 diagnosis trained on data from US electronic health records (IBM Explorys), including demographics, comorbidities, symptoms, and hospitalization. Out of 15753 COVID-19 patients, 2050 went into critical state or deceased. Non-random train-test splits by time were repeated 100 times and led to a ROC AUC of 0.861 [0.838, 0.883] and a precision-recall AUC of 0.434 [0.414, 0.485] (median and interquartile range). The interpretability analysis confirmed evidence on major risk factors (e.g., older age, higher BMI, male gender, diabetes, and cardiovascular disease) in an efficient way compared to clinical studies, demonstrating the model validity. Such personalized predictions could enable fine-graded risk stratification for optimized care management.

## Introduction

The coronavirus disease (COVID-19), caused by the severe acute respiratory syndrome coronavirus 2 (SARS-CoV-2)^1^, has started to spread since December 2019 from the province Hubei of the People’s Republic of China to 188 countries, becoming a global pandemic^2^. Despite having a lower case fatality rate than SARS in 2003 and MERS in 2012^3^, the overall number of 24,007,049 cases and 821,933 deaths from COVID-19^2^ (status August 26, 2020) far outweigh the other two epidemics. These high numbers have forced governments to respond with severe containment strategies to delay the spread of COVID-19 in order to avoid a global health crisis and collapse of the healthcare systems^4,5^. Several countries have been facing shortages of intensive care beds or medical equipment such as ventilators^6^. Given these circumstances, appropriate prognostic tools for identifying high-risk populations and helping triage are essential for informed protection policies by policymakers and optimal resource allocation to ensure best possible and early care for the patients.

Today’s availability of data enables the development of different solutions using machine learning to address these needs, as described in recent reviews^7,8^. One type of proposed solutions is prognostic prediction modeling, which consists in predicting patient outcomes such as hospitalization, exacerbation to a critical state, or mortality, using longitudinal data from medical healthcare records of COVID-19 patients^9–19^ or proxy datasets based on other upper respiratory infections^20^. To this date, most studies include data exclusively from one or few hospitals and therefore relatively small sample sizes of COVID-19 patients (i.e., below 1000 patients), with the exception of the retrospective studies in New York City with 4103^16^ or with a total of 3055 patients^17^.

This is where combined electronic health records (EHRs) across a large network of hospitals and care providers become valuable to generate real-world evidence (RWE), such as for the development of the 4C Mortality Score for COVID-19^21^. Machine learning models based on such datasets can benefit from increased amount of data and improved robustness and generalizability, as data comes from various sources (e.g., different hospitals), and may thus cover wider ranges of demographics and diverse healthcare practices or systems. Having such data available, can facilitate and accelerate insight generation, as such an approach for retrospective data analyses is more cost effective and requires less effort compared to setting up and running large-scale clinical studies. The IBM® Explorys® database (IBM, Armonk, NY) is one example for a large set of de-identified EHRs of 64 million patients across the US including patient demographics, diagnoses, procedures, prescribed drugs, vitals, and laboratory test results^22^. However, it is not possible to predict mortality using this dataset, as death is not reliably reported and the EHRs cannot be linked to public death records due to de-identification.

The aim of this work was to create a prognostic prediction model for critical state after COVID-19 diagnosis based on a retrospective analysis of a large set of de-identified EHRs of patients across the US using the IBM® Explorys® database (IBM, Armonk, NY). Such a predictive model allows identifying patients at risk based on predictive factors to support risk stratification and enable early triage. The present work based on EHR data is reported according to the RECORD and STROBE statements^23^, and reporting of model development followed TRIPOD statement guidelines^24^.

## Results

### Cohort, descriptive statistics, and concurvity

**Fig. 1:**
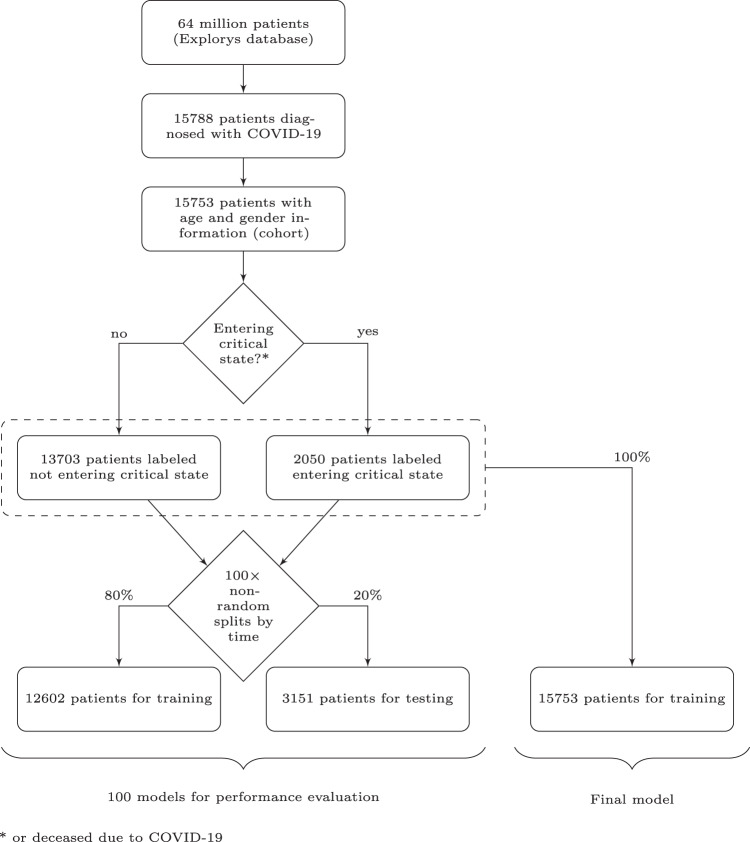
Diagram of number of subjects. Cohort selection and number of patients not entering versus entering critical state based on the definitions outlined in the according sections. To train and evaluate a model, the dataset was split using a non-random split by time. This procedure was repeated 100 times using different time windows for the test sets to get a distribution of model performance.

**Fig. 2:**
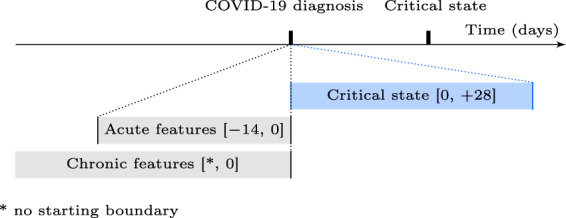
Time windows for prediction target and feature extraction. Schematic illustration of time window definitions relative to the COVID-19 diagnosis or to the critical state (time not to scale). The brackets define the boundaries (included) in days.

The total number of identified patients diagnosed with COVID-19 based on International Classification of Diseases (ICD) codes and entries for positive results of SARS-CoV-2 tests based on Logical Observation Identifiers Names and Codes (LOINC) are reported in Fig. 1. Patients without either age or gender information were subsequently removed, and the remaining patients are referred to as the cohort in the present manuscript. For the binary prediction, patients were labeled either as not entering critical state (*N* = 13,703) or as entering critical state (*N* = 2050). Entering critical state encompassed patients with a reported ICD code for sepsis, septic shock, or respiratory failure (e.g., acute respiratory distress syndrome (ARDS)) within the 28 days after COVID-19 diagnosis (Fig. 2), or patients being flagged as deceased in the database without having been in a critical state before COVID-19 diagnosis. Moreover, the sizes of the partitions for training and testing are also reported in Fig. 1. Among patients labeled as critical state, a total of 545 patients were flagged as deceased in the Explorys database. This corresponds to 3.5% of the entire cohort. There were 11 cases of deceased patients without critical state after COVID-19 diagnosis, which represent less than 0.1% of the cohort.

**Fig. 3:**
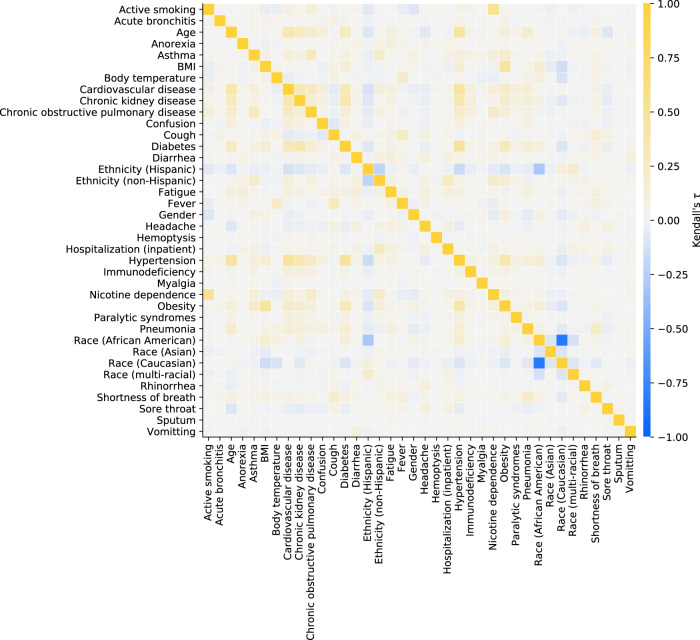
Feature concurvity. Kendall’s *τ* was used to evaluate correlation between each feature combination (prior to feature exclusion in feature set pre-processing).

The set of considered features consisted in demographics, “acute” features (mostly symptoms potentially related to COVID-19), and “chronic” features (mostly comorbidities or behaviors previously acquired and not related to COVID-19), extracted according to Fig. 2. Symptoms and comorbidities, in particular, were represented as binary features (1: ICD code entry exists in database; 0: no entry recorded for the specific patient). Details for ICD and LOINC codes are listed in Table 1. Descriptive statistics for all features are reported in Table 2. No features were removed due to a too high proportion of missing data. Rank correlations across features are shown in the heatmap in Fig. 3. As Caucasian and African American together represent over 90% of the dataset, keeping both features race (Caucasian) and race (African American) is unnecessary, as they encode almost the identical information content, for which reason the majority group (i.e., race (Caucasian)) was removed from the feature set and contributes to the baseline risk probability. No other features were removed due to high feature collinearity. The resulting feature set is later referred to as full feature set.

**Table 1:** Feature definitions.

Extraction time window	Feature	Units	Details
Special features	Age	Years	Computed at diagnosis date, based on birth year entry
	Gender	NA (0: male, 1: female)	No time window restrictions
	Ethnicity (Hispanic)	NA (binary)	No time window restrictions
	Ethnicity (non-Hispanic)	NA (binary)	No time window restrictions
	Race (African American)	NA (binary)	No time window restrictions
	Race (Asian)	NA (binary)	No time window restrictions
	Race (Caucasian)	NA (binary)	No time window restrictions
	Race (Multiracial)	NA (binary)	No time window restrictions
Acute features	Acute bronchitis	NA (binary)	ICD-10: J20.*, J40 and ICD-9: 466.0, 490
	Anorexia	NA (binary)	ICD-10: R63.0, R63.8 and ICD-9: 783.0, 783.9
	Body temperature	^∘^C	LOINC: 8310-5
	Confusion	NA (binary)	ICD-10: R41.0, R41.82 and ICD-9: 780.97
	Cough	NA (binary)	ICD-10: R05 and ICD-9: 786.2
	Diarrhea	NA (binary)	ICD-10: R19.7 and ICD-9: 787.91
	Fatigue	NA (binary)	ICD-10: R53.1, R53.81, R53.83 and ICD-9: 780.79
	Fever	NA (binary)	ICD-10: R50.9 and ICD-9: 780.60
	Headache	NA (binary)	ICD-10: R51 and ICD-9: 784.0
	Hemoptysis	NA (binary)	ICD-10: R04.2 and ICD-9: 786.30
	Hospitalization (inpatient)	NA (binary)	Considered if reported admission–discharge period overlapping with extraction time window
	Myalgia	NA (binary)	ICD-10: M79.1, M79.10, M79.11, M79.12, M79.18 and ICD-9 729.1
	Pneumonia	NA (binary)	ICD-10: J12.*, J13, J14, J15.*, J16.*, J17, J18.* and ICD-9: 480.*, 481, 482.*, 483.*, 484.*, 485, 486, 487.0, 488.01, 488.11, 488.81
	Rhinorrhea	NA (binary)	ICD-10: J34.89 and ICD-9: 478.19
	Shortness of breath	NA (binary)	ICD-10: R06.02 and ICD-9: 786.05
	Sore throat	NA (binary)	ICD-10: J02.9 and ICD-9: 462
	Sputum	NA (binary)	ICD-10: R09.3 and ICD-9: 786.4
	Vomiting	NA (binary)	ICD-10: R11.10 and ICD-9: 536.2, 787.03
Chronic features	Active smoking	NA (binary)	Based on reported habit
	Asthma	NA (binary)	ICD-10: J45.* and ICD-9: 493.*
	BMI	kg/m^2^	LOINC: 39156-5, or computed from weight (29463-7) and height (8302-2)
	Cardiovascular disease	NA (binary)	ICD-10: I20.*, I21.*, I25.*, I48.*, I50.*, I63.*, I65.*, I67.*, I73.* and ICD-9: 410.*, 412.*, 413.*, 414.*, 427.*, 428.*, 429.*, 433.*, 434.*, 437.*, 443.*
	Chronic kidney disease	NA (binary)	ICD-10: E10.21, E10.22, E10.29, E11.21, E11.22, E11.29, I12.0, I12.9, I13.0, I13.10, I13.11, I13.2, N04.*, N05.*, N08, N18.*, N19, N25.9 and ICD-9: 250.40, 250.41, 250.42, 250.43, 403.*, 404.*, 581.81, 581.9, 583.89, 585.*, 588.9
	Chronic obstructive pulmonary disease	NA (binary)	ICD-10: J44.* and ICD-9: 491.*, 493.2*
	Diabetes	NA (binary)	ICD-10: E10.*, E11.*, E13.* and ICD-9: 250.*
	Hypertension	NA (binary)	ICD-10: I10, I15.* and ICD-9: 401.*, 405.*
	Immunodeficiency	NA (binary)	ICD-10: B20, D80.*, D81.*, D82.*, D83.*, D84.*, D86.*, D89.* and ICD-9: 042, 279.*
	Nicotine dependence	NA (binary)	ICD-10: F17.* and ICD-9: 305.1
	Obesity	NA (binary)	ICD-10: E66.0*, E66.1, E66.2, E66.8, E66.9 and ICD-9: 278.00, 278.01, 278.03
	Paralytic syndromes	NA (binary)	ICD-10: G80.*, G81.*, G82.*, G83.* and ICD-9: 342.*, 343.*, 344.*

Feature names, units and details (e.g., ICD and LOINC codes) grouped by extraction time window specifications. Binary features encode whether there is an entry in the database for the specific item of interest or not. The symbol * represents a wildcard for ICD subcategory codes.

**Table 2:** Descriptive statistics of the features.

Feature	Missing	Mean	Std	Min	25%	50%	75%	Max
Age	NA	48.6	19.4	1	32	49	63	90
BMI	16.7%	31.6	8.41	10.4	25.7	30.1	36	93
Body temperature	69.3%	37.1	0.617	32	36.7	37	37.4	40.8
**Feature**	**Missing**	**Female (1)**	**Male (0)**					
Gender	NA	56.9%	43.1%					
**Feature**	**Present (1)**	**Absent (0)**						
Active smoking	16.0%	84.0%						
Acute bronchitis	1.5%	98.5%						
Anorexia	0.9%	99.1%						
Asthma	11.4%	88.6%						
Cardiovascular disease	23.4%	76.6%						
Chronic kidney disease	10.0%	90.0%						
Chronic obstructive pulmonary disease	5.6%	94.4%						
Confusion	1.8%	98.2%						
Cough	29.9%	70.1%						
Diabetes	18.8%	81.2%						
Diarrhea	4.1%	95.9%						
Ethnicity (Hispanic)	11.6%	88.4%						
Ethnicity (non-Hispanic)	29.3%	70.7%						
Fatigue	7.4%	92.6%						
Fever	22.2%	77.8%						
Headache	4.9%	95.1%						
Hemoptysis	0.1%	99.9%						
Hospitalization (inpatient)	4.2%	95.8%						
Hypertension	38.0%	62.0%						
Immunodeficiency	2.3%	97.7%						
Myalgia	0.2%	99.8%						
Nicotine dependence	8.6%	91.4%						
Obesity	25.0%	75.0%						
Paralytic syndromes	1.2%	98.8%						
Pneumonia	12.2%	87.8%						
Race (African American)	44.2%	55.8%						
Race (Asian)	1.2%	98.8%						
Race (Caucasian)	48.5%	51.5%						
Race (multi-racial)	2.3%	97.7%						
Rhinorrhea	1.7%	98.3%						
Shortness of breath	15.4%	84.6%						
Sore throat	3.7%	96.3%						
Sputum	0.0%	100.0%						
Vomitting	0.6%	99.4%						

The descriptive statistics are reported for all features (prior to feature exclusion in feature set pre-processing). The percentages 25, 50, and 75% refer to the first (Q1), second (median), and third quartiles (Q3). Note that as part of Explorys’ de-identification process the feature age has a ceiling effect at 90 years, and the age of all patients born in the last 365 days is reported as zero. The features age and gender were mandatory based on a previous data preparation step, hence the rate of missing data is not reported for these features.

### Performance

**Fig. 4:**
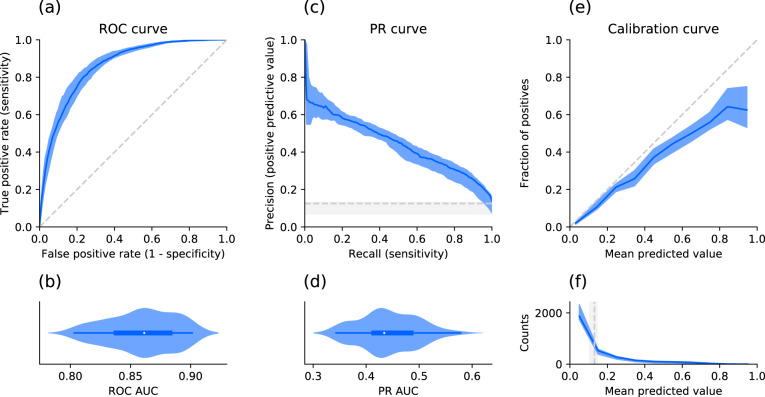
Model performance and calibration for the reduced feature sets across splits. **a** Receiver operating characteristic (ROC) curve: Median and interquartile range (IQR) of the performance (blue) and chance level (no predictive value) as a reference (dashed gray line). **b** Corresponding normalized violin plot of the distribution of the ROC area under the curve (AUC). **c** Same representation for the precision recall (PR) curve and **d** corresponding distribution of the PR AUC. **e** Median and IQR (blue) of the fraction of actual positives (labeled as critical state) for the binned (10 bins) mean predicted values (i.e., probabilities). The reference diagonal represents perfect calibration (dashed gray line). **f** Median and IQR (blue) for the counts within each bin of mean predicted value. The vertical gray line shows the median and IQR for the optimal decision threshold based on the Youden’s J statistic.

**Fig. 5:**
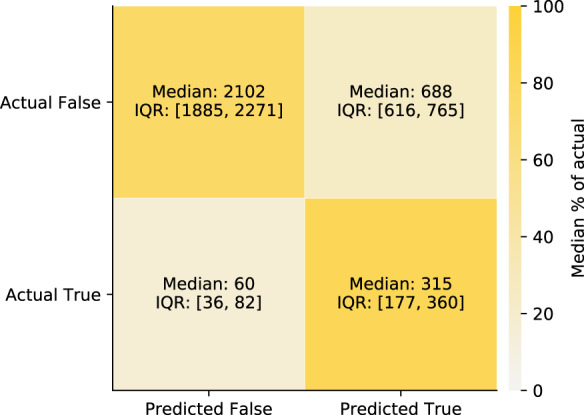
Confusion matrix for the reduced feature sets across splits. Confusion matrix for the predictions of the test sets based on the optimal decision threshold based on the Youden’s J statistic. True refers to entering critical state, and False refers to not entering critical state. The shades of the confusion matrix correspond to the median percentage of the actual labels (i.e., shade of the top left cell and the bottom right cell represent the median specificity and the median sensitivity, respectively).

To obtain a distribution of prediction performance, 100 non-random train-test splits by time of the dataset were created to train and evaluate 100 individual XGBoost models. This was done once on the full feature set (excluding features removed in the data preparation step), and in a second step on split-specific reduced feature sets keeping only the most relevant features based on a feature importance analysis in each split in order to simplify the models. The performances of the full and reduced models are summarized using different metrics in Table 3. Figure 4a–d shows the receiver operating characteristic (ROC) and the precision-recall (PR) curves together with their distributions of their areas under the curve (AUC) as well as the calibration of the models (e) for the 100 models after feature reduction. In addition, the confusion matrix for the identified optimal classification threshold (0.131 [0.105, 0.146]) is shown in Fig. 5. The sensitivity of the models for the optimal threshold was 0.829 [0.805, 0.852] and the specificity 0.754 [0.713, 0.785].

**Table 3:** Performance comparison.

Performance metric	Full feature set	Reduced feature set
ROC AUC	0.863 [0.838, 0.885]	0.861 [0.838, 0.883]
PR AUC	0.443 [0.405, 0.489]	0.434 [0.414, 0.485]
Brier score	0.081 [0.050, 0.095]	0.082 [0.050, 0.095]
Log loss	0.265 [0.177, 0.305]	0.269 [0.178, 0.306]
Sensitivity	0.828 [0.805, 0.855]	0.829 [0.805, 0.852]
Specificity	0.749 [0.702, 0.786]	0.754 [0.713, 0.785]
*F*1-score	0.439 [0.374, 0.475]	0.439 [0.368, 0.478]

Different test set performance metric distributions across splits for the models before and after feature reduction based on feature importance. Numbers are reported as median and interquartile range.

### Feature reduction and model interpretability

**Fig. 6:**
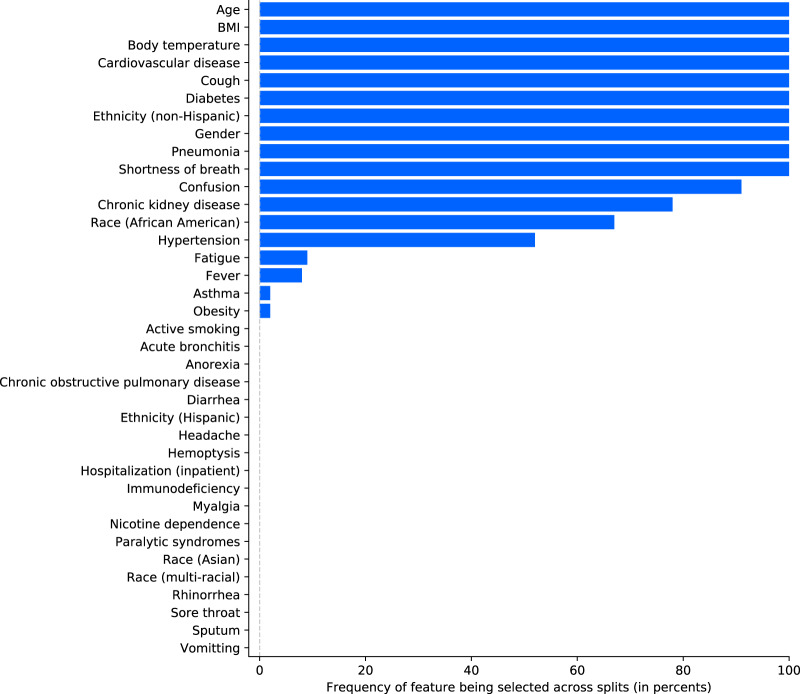
Feature selection frequency across splits. Features are first ordered by frequency of being selected during the feature selection process of each split and then alphabetically in case of identical frequency.

**Fig. 7:**
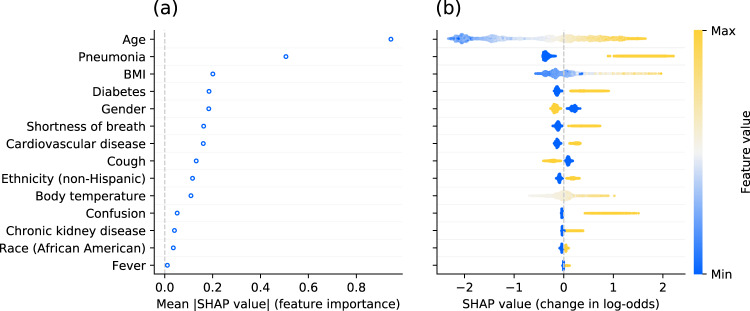
Final model interpretability for the reduced feature set. **a** Average absolute impact of features on the final model output magnitude (in log-odds) ordered by decreasing feature importance. **b** Illustration of the relation between feature values and impact (in terms of magnitude and direction) on prediction output. Each dot represents an individual patient of the dataset. The color of each point corresponds to the normalized feature value (min-max normalization on test set). As an example for continuous features, older patients tend to have a higher SHAP value. For binary features, the maximum feature value 1 corresponds to presence of the feature, and 0 to absence of the feature. For gender, 1 corresponds to female and 0 to male.

**Fig. 8:**
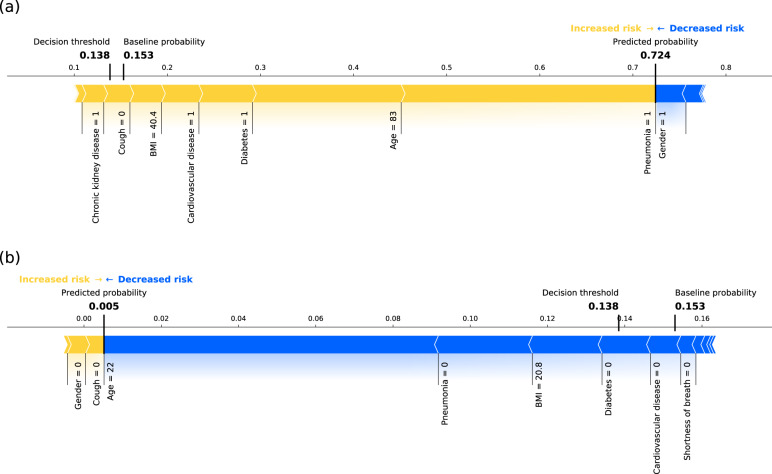
Example predictions using the final model. Composition of predictions (SHAP values computed in probability space) for **a** one example patient going into critical state and **b** one patient not entering critical state, based on the final model. The yellow arrows represent the contributions of major risk factors (e.g., older age, pneumonia, or comorbidities), and the blue arrows represent the contribution of factors decreasing the probability of entering critical state (e.g., female gender = 1). Note that this graphical representation only allows due to spatial constraints to display the names of the major contributing factors for this specific example (i.e., a subset of all features used by final model). The baseline probability and the decision threshold are relatively low but close to the actual ratio of patients entering critical state in our cohort, illustrating the class imbalance.

Figure 6 shows the results of the feature reduction process in terms of frequency of a feature being selected across the 100 splits based on its mean absolute SHAP (SHapley Additive exPlanations^25^) value. Most features were either always or never selected, demonstrating high homogeneity across different splits. Figure 7 shows the results of the model interpretability analysis based on Tree SHAP^25^ for the final model fitted using the same methodology as for the 100 models after feature reduction, but trained on all patient records to maximize the use of information. Older age and pneumonia are by far the principal predictors for critical state. The features contributing to a higher probability of critical state in case of high feature values or presence are (in decreasing order of global feature importance): older age, pneumonia, higher BMI, diabetes, male gender, shortness of breath, cardiovascular disease, absence of cough, non-Hispanic ethnicity, higher body temperature, confusion, chronic kidney disease, race (African American), and fever. Note that in Fig. 7 for binary features "max” feature values correspond to 1 (e.g., presence of the feature). In the case of gender, 1 corresponds to female (see Table 1). Figure 8 illustrates the composition of two example predictions from the final model.

## Discussion

In this work, a prognostic model was created based on real-world data to predict at COVID-19 diagnosis, whether patients will enter a critical state within the next 28 days or not. Our results from 100 non-random train-test splits by time (12,602 patients for training and 3151 patients unseen during training for testing) showed high predictive performance (median sensitivity of 0.829 and specificity of 0.754) and acceptably-calibrated output probabilities with a minor tendency to over-forecast probabilities. Furthermore, the interpretability analysis identified older age, pneumonia, higher BMI, diabetes, male gender, shortness of breath, cardiovascular disease, absence of cough, non-Hispanic ethnicity, and higher body temperature as most important predictive factors for critical state.

Around 16,000 US patients diagnosed with COVID-19 met the inclusion criteria. To the best of our knowledge, it is one of the largest cohorts used for COVID-19 progression modeling to date based on EHR data. The definitions used for severe state or critical state vary across different sources (e.g., intubation prior to ICU admission, discharge to hospice, or death^17^, moderate to severe respiratory failure^11^, oxygen requirement greater than 10 L/min or death^13^), or are not described in detail. Based on the definition by the World Health Organization^26^ including sepsis, septic shock, and respiratory failure (e.g., acute respiratory distress syndrome (ARDS)), the proportion of patients entering critical state (13.0%) in our study is within the range of prevalence (12.6–23.5%) reported in a review covering 21 studies^27^. Similarly, case fatality rates vary across US states and countries, as they directly depend on factors such as the number of tested people, demographics, socioeconomics, or healthcare system capacities. The death rate for the entire US is estimated to be 3%^2^ (status August 26, 2020). In the present work, the reported proportion of people assumed to be deceased because of COVID-19 is 3.5%. These differences may be justified in part by the fact that in these sources the outcome (i.e., potential death) of recent cases is yet unknown when computing the case fatality rate, hence leading to underestimation. As our analysis enforces at least 7 weeks of data after diagnosis date increasing changes of knowing the patients’ outcomes, this underestimation can be reduced. Nevertheless, death is not reliably reported in EHRs and records were de-identified making linking to public death records not feasible. Regarding demographics of our cohort, there are only minor dissimilarities to numbers reported by the Centers for Disease Control and Prevention (CDC) or US states. The interquartile range of the age distribution of our cohort (32–63 years) matches with the 33–63 years for COVID-19 cases across the entire US^28^. The racial breakdown varies strongly across different US states. Given that Explorys clients are mostly in metropolitan areas, there is a higher proportion of African Americans in the present EHR dataset compared to US average^29^. The proportion of female cases (56.9%) is more pronounced compared to the US-wide incidences of 406 (female) and 401 (male) cases per 100,000 persons also showing a marginally higher rate for females than males, respectively^28^. The most common underlying comorbidities identified through ICD codes in our cohort are hypertension, obesity, cardiovascular disease, diabetes, and chronic lung disease (includes asthma and chronic obstructive pulmonary disease). As this is in line with statistics from the CDC^28,30^ as well as other studies conducted in China (e.g.,^31^) and the prevalence of such features is not affected by any time window restrictions (i.e., the entire patient history was considered), it substantiates the validity of the Explorys data. Since the aim of the present work is to develop a model for predictions at the time point of COVID-19 diagnosis, symptoms identified through ICD codes (e.g., fever or cough) are only extracted from the 14 days previous to the COVID-19 diagnosis. As the COVID-19 diagnosis may be early or late in the disease progression, there is the possibility to capture either early or late symptoms depending on each case. However, due to the time window restriction, the prevalence of reported symptoms tends to be lower compared to statistics including reported symptoms during the entire course of the disease^28^. Moreover, outpatient symptoms based on ICD codes may be under-documented, as hospitals may not get paid for their diagnosis. In spite of these lower numbers, the most common symptoms in our cohort, namely cough, fever, and shortness of breath, are confirmed by other reports and studies^28,32,33^. In summary, despite the high sparsity of Explorys EHR data, the size and quality of the extracted dataset demonstrates high value and validity for the present use case.

Although our dataset is based on sparse real-world data, our prognostic model shows an excellent model performance in terms of ROC AUC (0.861 [0.838, 0.883])^34^ and a substantial improvement of the PR AUC (0.434 [0.414, 0.485]) compared to chance level (0.125 [0.068, 0.133]). While the optimal decision threshold for a medical application may differ from the threshold based on the Youden’s J statistic, as in some applications high sensitivity and in others high specificity is more important, the Youden’s J statistic allows creating a scenario with equally weighted sensitivity and specificity for comparing models using the same optimization criteria. Maximizing the Youden’s J statistic (0.575 [0.537, 0.622]) leads to a sensitivity of 0.829 [0.805, 0.852] and a specificity of 0.754 [0.713, 0.785]. For this example decision threshold and sensitivity one obtains a median precision (or positive predictive value, PPV) of 0.296 (see Fig. 4). The PPV describes the percentage of patients actually entering critical state when they are predicted to enter critical state, i.e., 29.6% of the cases (compared to the chance level of 12.5 [6.8, 13.3]). While this seems rather low, it may depend in what setting the model is used. If medical resources are scarce, a model with a low false positive rate and high PPV, and thus with a lower sensitivity, would be favorable. Hence, the decision threshold should be increased. In that case, with for example a specificity of 97.5%, the PPV would be 56.2%. Furthermore, the results in Table 3 demonstrate that the feature reduction based on feature importance does not impact the model performance, yet simplifies the models. As different types of datasets, inclusion/exclusion criteria, features, and prediction target definitions were used in other papers presenting the development of models predicting COVID-19 critical state, (e.g.,^13,17^, or review^8^), it renders it difficult to do a direct performance comparison (reported metrics^11,13,17,35^ were in the following ranges: ROC AUC 0.81–0.99, PR AUC 0.56–0.71, sensitivity 0.70–0.94, specificity 0.74–0.96). Furthermore, some publications do not mention some metrics (e.g., PR AUC), which are complementary and particularly useful for imbalanced datasets, as focusing only on high ROC AUC values (potentially resulting from high class imbalance) may lead to overoptimistic interpretation of model performance^36^. Unlike other papers^11,13,17^ usually performing a cross-validation or using a limited number of independent sets for the testing, the present approach used non-random train-test splits by time repeated 100 times to obtain a distribution of performance. Such an approach has the advantage of providing a better understanding of the generalizability of the model and the robustness of the performance estimate, as it is likely that a single test set might underestimate or overestimate the real performance for small testing sets. Even though our model was trained on data coming from many hospitals compared to other work being only based on a single or limited number of contributors, an external validation should be performed to better assess its generalizability. Most publications on prognosis prediction models do not report model calibration^8^, with the exception of a few^13,18^. The present model based on the Explorys dataset is acceptably-calibrated, showing only minor tendency to over-forecast probabilities. This over-forecast at higher predicted probabilities may be due to a low percentage of cases with critical state. In any case, over-forecast accentuating cases with relatively high probability is preferable to under-forecast, where patients with high probability of critical state may not be identified. Overall, our prognostic model shows excellent performance and has the advantage to provide an acceptably-calibrated risk score instead of a binary classification. This could potentially help healthcare professionals to create a more fine-graded risk stratification of patients.

Pneumonia appeared among the top features, as pneumonia is a diagnosis defining moderate and severe cases^26^, which are precursor stages for critical state due to COVID-19 disease. The results from a study with 1099 patients showed that patients with severe disease had a higher incidence of physician-diagnosed pneumonia than those with non-severe disease^37^. As identified by the interpretability analysis, older age is an important risk factor. This has been confirmed by many studies showing its relevance in progressing to grade IV and V on the pneumonia severity index and mortality of COVID-19 patients^38–40^. The developed model was also able to endorse existing results showing that men are, despite similar prevalence to women, more at risk for worse disease severity, independent of age^41^. Similarly, obesity has been reported as a factor increasing probability of higher disease severity and lethality^37,42,43^. While the feature obesity shows minimal importance, the feature BMI is among the top features leading to high risk (in case of high BMI). It can be assumed that the feature obesity with a prevalence of 25.1% in our dataset compared to age-adjusted prevalence of obesity in the US is around 35%^44^ is under-reported in the EHR data of our cohort. The median BMI in our dataset is very close to the threshold from overweight to obesity (BMI of >30 kg/m^2^). Hence it can be concluded that approximately 50% of our patients are obese. In addition, the BMI feature is a continuous variable with only 16.7% missing entries, having thus more information content and, as a result, shows higher predictive importance than obesity. In line with the literature, the following comorbidities were also shown to drive high probabilities for critical state: diabetes^45–47^, chronic kidney disease^48–50^, and cardiovascular diseases^40,51,52^. As a matter of fact, many elderly patients with these comorbidities use Angiotensin-converting enzyme (ACE) inhibitors and Angiotensin-receptor blockers (ARBs), which upregulate the ACE-2 receptor^53^. Given that ACE-2 receptor has been proposed as a functional receptor for the cell entry mechanism of coronaviruses, it has been hypothesized that as a consequence this may lead to a higher prevalence and elevated risk for a severe disease progression after SARS-CoV-2 infection^54^. The two primary symptoms influencing the progression of the disease based on the present analysis are shortness of breath (dyspnea) and cough, both prevalent symptoms for COVID-19^33^. Interestingly, they have opposite effects on the prediction probability of the model, with shortness of breath increasing and cough decreasing the probability for critical state. This can be explained by the fact that cough is an early symptom during mild or moderate disease, and shortness of breath develops in the late course of illness. This concurs with statistical reports from China showing higher prevalence of shortness of breath in severe cases and a higher prevalence of cough in non-severe cases and survivors^31,55,56^. Hence, if cough is reported, this may indicate that the disease is still in early stage and there is the chance that it may not lead to a critical state, whereas if shortness of breath is reported, chances for further disease progression may be much higher. Furthermore, hospitals may not report outpatient symptoms such as cough, whereas they may report more critical symptoms such as shortness of breath more reliably. This means that it is highly likely that many of the patients in our cohort without an ICD code entry for cough actually may have had cough, in particular given that it is a highly prevalent symptom. This may considerably contribute to this rather surprising result. High body temperature also emerges as an important feature for predicting critical state. Despite having a high level of missing data, it appears to have more value given its continuous scale compared to the binary feature fever, similarly to the case of BMI vs. obesity. Nonspecific neurological symptoms like confusion are less commonly reported^32^. Nevertheless, the plot (b) in Fig. 7 reveals that the presence of confusion significantly contributes to an increase in the model’s output probability, despite having low overall importance (which in turn is also driven by the low prevalence within our dataset). Confusion may be a clearer precursor of neuroinvasion of SARS-CoV-2, which has been suggested to potentially lead to respiratory failure^57^. Overall, the findings of this work are in line with results from the vast number of studies reported in the literature and the interpretability analysis provides evidence for the validity of the prognostic prediction modeling. When comparing the most relevant features in the present work with models predicting comparable disease progression, there are some similarities but also major differences: Some models include mostly laboratory biomarkers and other vital measurements^13^, while others also include for example comorbidities^17^ and signs and symptoms^11^. The feature importance analysis of a model including both biomarkers and comorbidities revealed that the most important features are biomarkers^17^. Similarly, different combinations of types of features were explored, showing that the performance in terms of ROC AUC is higher when using laboratory biomarkers compared to signs and symptoms and that there is only a marginal improvement when including comorbidities as additional covariates^11^. Interestingly, our model shows similar classification performance as the models above, despite not including biomarkers and having the features being restricted to only demographics, comorbidities, and signs and symptoms. Given the fact that the model of this present work does not rely on biomarkers, it increases its applicability.

EHRs can be a powerful data source to create evidence based on real-world data, especially when combined with a platform facilitating the structured extraction of data. However, there are trade-offs to be made when doing analyses on EHR data in contrast to the analysis of clinical study data^58^. One major limitation is that patients may get diagnoses, treatments, or observations outside of the hospital network covered by Explorys, resulting in sparse patient histories. Other challenges are potential over- and under-reporting of diagnoses, observations, or procedures. For example, clinicians may enter an ICD-10 code for COVID-19 when ordering a SARS-CoV-2 test leading to over-documentation and “false positive” entries. On the other hand, relying only on test results may increase the risk that tested patients only performed the test at a hospital within the Explorys network, but did not get diagnosed and treated within the same hospital, which would lead to potentially “false negatives” in terms of target labeling. For this reason the inclusion criteria for our cohort was based on the combination of an ICD code entry for COVID-19 with a positive SARS-CoV-2 test result, to increase the probability of only including patients with actual COVID-19. EHR data often requires imputation, as there is rarely a patient with a complete data record, especially when the set of features is large. The method of imputation may also introduce additional biases which are difficult to control. One limitation of binary features encoding presence or absence of entries in an EHR system (e.g., for comorbidities) is that in case of patients without an entry it cannot be known whether the patient does not suffer from this condition or whether the patient does suffer from this condition, but it has not been diagnosed or reported in this EHR. Therefore, in the present work, the model has to rely on whether this information is available or not. In the other cases (e.g., body temperature or BMI), it was ensured that the imputation was based purely on the train set to avoid information leakage, which is particularly important in predictive modeling. Furthermore, to ensure data privacy and prevent re-identification, patients’ age is truncated, and death dates and related diagnoses and procedures are not available in Explorys data. As the latter is relevant for the present modeling, several assumptions had to be taken. For example in the 11 cases of deceased patients without critical state after COVID-19 diagnosis, it was assumed that they deceased due to COVID-19. However, they may have also deceased due to another reason. Nevertheless, as they represent less than 0.1% of our cohort, this assumption does not substantially influence the modeling. Furthermore, resulting death rates correspond well to official COVID-19-related death rates in the US or relevant states. An additional limitation and potential bias is linked to the data extraction using time windows. Even though the window lengths were motivated by medical reasoning, they are subject to trade-offs which is not the case for clinical studies due to precise protocols: extending the windows to capture enough information spread over multiple visits and account for delays in EHR entries, versus remaining recent enough and related to COVID-19. Furthermore, the features used in this model do not capture the time information for the individual samples (e.g., how many days before COVID-19 diagnosis the ICD code for fever was entered into the system). In addition, it could be that the reference for the time windows is not accurate, as the ICD code or LOINC entry used as COVID-19 diagnosis proxy may not have been the actual first diagnosis of the patient. The model was based on US data from hospitals of the Explorys network, sampling mostly metropolitan areas, resulting for example in a higher ratio of African Americans compared to the US average. Therefore it is highly likely that there are socioeconomic and demographic biases. Moreover, the data reflects the American healthcare system in terms of testing, diagnosing, and treating procedures as well as reporting. Thus, one major limitation of this work is the lack of external validation using a different dataset. Despite these limitations, RWE can retrospectively generate insights on a scale, which would not be feasible with an observational clinical study. Furthermore, approaches based on RWE might even have higher clinical applicability due to their incorporation of statistical noise while model training^59^.

The results of this work demonstrate that it is possible to develop an explainable machine learning model based on patient-level EHR data to predict at the time point of COVID-19 diagnosis, whether individual patients will progress into critical state in the following 4 weeks. Without the necessity of relying on multiple laboratory test results or imaging such as computer tomography, this model holds promise of clinical utility due to the simplicity of the relevant features and its adequate sensitivity and specificity. Even though this prognostic model for critical state has been trained and evaluated on one of the largest COVID-19 cohorts to date with EHR data from around 16,000 patients, it includes predominantly cases from metropolitan areas within the US and may therefore be biased towards sub-populations of the US and the American healthcare system. To prove its generalizability before being considered for clinical implementation, it should be validated with other datasets. Such RWE models have the potential to identify new risk factors by mining EHRs. This model could also be augmented with treatment features (e.g., drugs or other interventions) after diagnosis in order to predict whether the respective treatments would lead to an improvement (i.e., reduction of the probability of entering critical state). RWE approaches will never replace clinical studies to validate risk factors or evaluate treatment effectiveness. Nevertheless, these types of retrospective real-world data analyses can support other research generally requiring much higher efforts and costs: They could help identifying high risk or responder groups or informing the design of clinical trials, with the aim of making research more efficient and accelerating the avenue to personalized treatment and eventually reduced burden on the healthcare system.

## Methods

### RWE insights platform

This work was achieved by using the *RWE Insights Platform*, a data science platform for analyses of medical real-world data to generate RWE recently developed by IBM. The *RWE Insights Platform* is a data science pipeline facilitating the setup, execution, and reporting of analyses of medical real-world data to discover RWE insights in an accelerated way. The platform architecture is built in a fully modular way to be scalable to include different types of analyses (e.g., treatment pathway analysis, treatment response predictor analysis, comorbidity development analysis) and interface with different data sources (e.g., the Explorys database).

For the present use case of COVID-19 prognosis prediction, we used the comorbidity development analysis which allows defining a cohort, an outcome to be predicted, a set of predictors, and relative time windows for the extraction of the samples from the data source. New data-extraction modules for specific disease, outcome, treatments, and variables for the current use case were developed.

The *RWE Insights Platform* has been developed using open-source tools and includes a front end based on HTML and CSS interfacing via a Flask RESTful API to a Python back end (python 3.6.7) using the following main libraries: imbalanced-learn 0.6.2, numpy 1.15.4, pandas 0.23.4, scikit-learn 0.20.1, scipy 1.1.0, shap 0.35.0, statsmodel 0.90.0, and xgboost 0.90. The platform is a proprietary software owned by IBM. The detailed description of the *RWE Insights Platform* is beyond the scope of this publication.

### Real-world data source

Our work was based on de-identified data from the Explorys database. The Explorys database is one of the largest clinical datasets in the world containing EHRs of clinical activity of around 64 million patients distributed across more than 360 hospitals in the US^22^. This dataset contains data on patients in all 50 US states who seek care in healthcare systems, which chose the IBM Enterprise Performance Management platform for their population and performance management and is not tied to particular insurers. Data were standardized and normalized using common ontologies, searchable through a Health Insurance Portability and Accountability Act (HIPAA)-enabled, de-identified dataset from IBM Explorys. Individuals were seen in multiple primary and secondary healthcare systems from 1999 to 2020 with a combination of data from clinical electronic medical records, health-care system outgoing bills, and adjudicated payer claims. The de-identified EHR data include patient demographics, diagnoses, procedures, prescribed drugs, vitals, and laboratory test results. Hundreds of billions of clinical, operational, and financial data elements are processed, mapped, and classified into common standards (e.g., ICD, SNOMED, LOINC, and RxNorm). As a condition of allowing the use of the de-identified data for research, these systems cannot be identified. The aggregated Explorys data were statistically de-identified to meet the requirements of 45 Code of Federal Regulations *§* 164.514(b), 1996 HIPAA, and 2009 Health Information Technology for Economic and Clinical Health (HITECH) standards. Business affiliation agreements were in place between all participating healthcare systems, and Explorys regarding contribution of EHR data to the Explorys Platform and the use of these de-identified data. The Explorys dataset does not include data from patients, who indicated at patient onboarding that they did not wish to have their data used for de-identified secondary use. Since the Explorys dataset consists of de-identified data for secondary use, the use of said dataset is not considered a human study and thus ethical approval was not required for the present work. The Explorys database has been proven to be useful in many retrospective data analyses for different applications (e.g., refs. ^60–64^). As data in Explorys is updated continuously, a view of the database was created and frozen on August 26, 2020 for reproducibility of this work.

### Cohort

The cohort included all patients in the Explorys database having a documented diagnosis of COVID-19 and a reported positive entry for a SARS-CoV-2 test, both since January 20, 2020. As the new ICD-10 code U07.1 for COVID-19 cases confirmed by laboratory testing has been created and pre-released a couple of months after pandemic onset, already existing ICD codes related to coronavirus (B34.2 Coronavirus infection, unspecified and B97.29 Other coronavirus as the cause of diseases classified elsewhere) were also included, as hospitals may have used them for early cases. Based on their appearance in Explorys, the following LOINC codes for polymerase chain reaction (PCR) tests for the detection of SARS-CoV-2 (COVID-19) RNA presence were included: 94309-2, 94500-6, and 94502-2 (see ref. ^65^ for detailed descriptions of the tests). The January 2020 cutoff was instituted to be consistent with the spread of COVID-19 in the US and to limit inclusion of patients, who may have been diagnosed with other forms of coronavirus besides SARS-CoV-2. In case of multiple entries per patient after January 20, 2020, the first ICD code or LOINC entry date was used as COVID-19 diagnosis date. In order to have enough data to extract the patient’s outcome, the diagnosis date had to be at least 7 weeks before the freeze date of the database (August 26, 2020), i.e., July 8, 2020, as it may take up to 7 weeks from symptom onset to death^66^.

### Prediction target

Critical state was used as a binary prediction target and included sepsis, septic shock, and respiratory failure (e.g., ARDS)^26^. Severe sepsis is associated with multiple organ dysfunction syndrome. The precise definition based on ICD codes used for critical state is listed in Table 4. In case of multiple entries for a patient, the first entry was retained. In addition, the date of the entry for critical state had to be in a window of [0, +28] days (boundaries included) after the diagnosis date to be eligible, as illustrated in Fig. 2. Four weeks were chosen to ensure coverage of the majority of critical outcomes, as the interquartile range of time from illness onset to sepsis and ARDS were reported to be [7, 13] and [8, 15] days, respectively^31^. Patients with an eligible entry for critical state were labeled as entering critical state, whereas patients eligible based on cohort definitions without any entry for critical state were labeled as not entering critical state. One exception to these rules were patients who are flagged as deceased in the Explorys database. In order to include death cases potentially related to COVID-19 in the critical state group, and as death dates and records with diagnoses and procedures relating to the patient’s death are not available in the Explorys data to avoid re-identification of patients and ensure data privacy, patients with one of the following conditions were also labeled as entering critical state: deceased with an entry for critical state within the window, deceased with an entry for critical state within and after the window, or deceased without any entry for critical state (and thus excluding deceased patients with an entry for critical state before the window). In the latter case, the date was set to the end of the window for critical state entries. To validate these assumptions, the proportion of patients assumed to be deceased due to COVID-19 in our cohort was compared to epidemiological numbers.

**Table 4:** ICD-10 codes for the prediction target.

ICD-10 code	Description
A41.89	Other specified sepsis
A41.9	Sepsis, unspecified organism
R65.2	Severe sepsis
R65.20	Severe sepsis without septic shock
R65.21	Severe sepsis with septic shock
J80	Acute respiratory distress syndrome (ARDS)
J96	Respiratory failure, not elsewhere classified
J96.0	Acute respiratory failure
J96.00	Acute respiratory failure, unspecified whether with hypoxia or hypercapnia
J96.01	Acute respiratory failure with hypoxia
J96.02	Acute respiratory failure with hypercapnia
J96.9	Respiratory failure, unspecified
J96.90	Respiratory failure, unspecified, unspecified whether with hypoxia or hypercapnia
J96.91	Respiratory failure, unspecified with hypoxia
J96.92	Respiratory failure, unspecified with hypercapnia

Patients with first diagnosis of any of the listed ICD-10 codes within the specified time window were labeled as entering critical state.

### Features

Features were mainly grouped into "acute” features and "chronic” features. Acute features are a set of features, which should be temporally close to the COVID-19 diagnosis (e.g., body temperature, symptoms potentially related to COVID-19, or hospitalization prior to the diagnosis), whereas chronic features are a set of features which have no direct temporal relation to the COVID-19 diagnosis (e.g., chronic comorbidities, measurable demographics, or long-term habits). Features were selected based on potential risk factors and predictors related to COVID-19 reported in the literature. Figure 2 illustrates their difference in terms of time windows for extraction. A negative value for boundaries of time window definitions stand for dates prior to the reference date (e.g., prior to the diagnosis date). Ideally, acute features should have been recorded for higher consistency at diagnosis date. However, this may not be always the case in the EHR compared to data from clinical studies. To account for recorded symptoms previous to the diagnosis (e.g., through telemedicine before performing a SARS-CoV-2 test, or due to potentially required multiple testing because of false negatives delaying diagnosis), a time window of [−14, 0] days before the diagnosis was used to extract acute features. Patients were considered hospitalized (inpatient) if the reported admission–discharge period of the hospitalization overlapped with the acute feature extraction time window. Following cases could occur: i) admission or discharge occurred within the acute feature extraction time window (start or end overlap), or ii) admission started before acute feature extraction time window and discharge occurred after the end of the window (complete overlap). Outpatients receiving a COVID-19 test or diagnosis at a hospital were not considered as hospitalized. Entries for chronic features were considered if prior to the diagnosis date, without additional restriction. Demographic features which were not restricted to any time window (e.g., gender or race) or required a special way of extraction/computation (e.g., age) are grouped as "special” features (see Table 1) and are not represented in Fig. 2. As part of the de-identification process, for patients over 90 years of age, the age is truncated to 90 years. Similarly, the age of all patients born within the last 365 days is set to 0 years. The full list of features including their definitions (e.g., based on ICD or LOINC codes) is provided in Table 1, grouped by extraction time window type. As features entries (especially relevant for chronic features) may have been entered several years ago, ICD-9 codes were used as well for the extraction. In general, the last entry within the specific extraction time window was used to construct the feature, except if described otherwise in Table 1. Binary features encode whether there is a reported entry in the database for the specific item of interest or not. Thus, in contrast to features representing actual values like body temperature, where no entry means missing information on body temperature they are by definition always either true (1) or false (0). As it is common for such features in EHR, not having an entry in the database (e.g., for a comorbidity) does not necessarily mean that the patient does not suffer from this comorbidity. Thus, patients not suffering from a condition and patients not being reported to suffer from a condition (latter case could be considered as “missing” data) are confounded. As features which are generally multivalue categorical variables (e.g., race) are represented as independent entries in Explorys, it can be the case that there are more than one and even conflicting entries in the database due to multiple and potentially erroneous reporting (e.g., there could be both an entry for Caucasian and for Asian). In order to fully reflect the information provided in the database, including these cases, and as the type of model used in the present work would require one-hot encoding of multivalue categorical variables, these features were implemented as independent binary variables.

### Dataset preparation, modeling, and evaluation

The full dataset was constructed based on COVID-19 diagnosis including binary prediction target labels for critical state and enriched by the various features. Patients with missing age or gender information were removed from the dataset. Descriptive distribution statistics were created for all features, and non-binary features with more than 90% missing values were removed from the feature set. For the remaining feature set, the concurvity (non-linear collinearity) among features was assessed using Kendall’s *τ*, a non-parametric measure of correlation. In case of ∣*τ*∣ > 0.7^67^, the feature with more missing values was removed from the feature set. In case both features had the same amount of missing values, the numeric feature with higher mean or the more frequent binary feature was removed. The latter allowed keeping minorities as features and embedding the majority into the baseline risk probability. To create a distribution and confidence intervals of the model performance, as performance may change depending on the choice of split, multiple non-random splits by time were created. The methodology of splitting by time is recommended in TRIPOD^68^, as it allows for non-random variation between the train and test sets, since all records of the test data of each split come from a time window which has not been seen during training of the respective split. For each split, the dataset was split into a train set (80%) and a test set (20%). A sliding window was applied on the chronologically ordered patients to create 100 different splits, where the window of 20% width corresponded to the test set of the split. Thus, for the first split, the test set covered the chronologically first 20% of the data records (earliest cases), while the test set of the 100th split corresponded to the last 20% (most recent cases). The remaining data of a split (whether before or after the test set window) was used as a train set.

For each non-random split by time the following steps were executed: The non-binary features of the train set and the test set were imputed based on multivariate feature imputation using Bayesian Ridge estimation fitted on the train set to avoid data leaking. This method imputes missing values by modeling each feature with missing values as a function of other features in a round-robin fashion^69,70^. The implementation of the iterative imputer of the scikit-learn package is based on the Multivariate Imputation by Chained Equations^71^ but returning a single imputation. As modeling approach, XGBoost, a decision-tree-based ensemble machine learning algorithm using a gradient boosting framework, was used. Gradient tree boosting models have shown to outperform other types of models on a large set of benchmarking datasets^72^. To tune the XGBoost hyperparameters, a five-fold cross-validation grid search on the training data maximizing the ROC AUC was used. Subsequently, an XGBoost model was re-trained on the entire train set using the previously identified parameters. The following grid of XGBoost parameters was evaluated: max_depth ∈ [2, 3, 4, 5, 6, 7, 8] (maximum depth of a tree), min_child_weight ∈ [1, 3, 5] (minimum sum of instance weight needed in a child), gamma ∈ [0.0, 0.1] (minimum loss reduction required to make a further partition on a leaf node of the tree), subsample ∈ [0.8, 1.0] (subsample ratio of the training instances), colsample_bytree ∈ [0.8, 1.0] (subsample ratio of columns when constructing each tree), reg_alpha ∈ [0.0, 0.01] (L1 regularization term on weights), and learning_rate ∈ [0.01, 0.05, 0.1] (step size shrinkage). The 100 trained XGBoost models were subsequently used to create predictions on the test set for performance evaluation.

The performance of the models was evaluated on the test set for each non-random train-test split by time and reported with median and interquartile range across different splits. This provides a distribution of expected performance, if a new model would be trained on similar data. Following metrics were computed: receiver operating characteristic (ROC) curve and precision recall (PR) curve as well as their respective areas under the curve (ROC AUC, also known as C-statistic, and PR AUC), Brier score, and log loss. The PR AUC is particularly useful to compare models from different datasets, which may be less or more imbalanced, as compared to the ROC AUC, the metric is not affected by class imbalance. The confusion matrix, sensitivity, specificity, and *F*1-score were reported for the optimal probability classification threshold. This threshold was obtained based on maximizing the largest Youden’s J statistic (corresponding to the largest geometric mean as a metric for imbalanced classification seeking for a balance between sensitivity and specificity). Furthermore, the calibration of the models was reported, comparing binned mean predicted values (i.e., probabilities) to the actual fraction of positives (labeled as critical state)^73^, in order to evaluate whether the predicted probability is realistic and can provide some confidence on the prediction.

Interpretability of the models was generated using Tree SHAP^25^, a version of SHAP (SHapley Additive exPlanations) optimized for tree-based models. SHAP is a framework to explain the contribution of feature values to the output of individual predictions by any type of model and to compute the global importance of features. This individual contribution is expressed as SHAP value, corresponding to log-odds (output of the trees in XGBoost). In order to reduce the complexity of the models by removing less important features (and therefore increasing the model’s applicability and reducing its dependency on imputation in cases where less feature values are available for a prediction), the mean absolute SHAP value was computed for each feature in each split. For each split, the features were ordered by decreasing mean absolute SHAP value and only the features representing when combined 95% of the sum of the mean absolute SHAP values were retained, thus removing the least important features which combined contribute to less than 5%. While this approach prevents leakage, this split-specific feature reduction process may result in a different reduced feature sets for the different splits. This approach is inspired by^74,75^ suggesting to use SHAP values for feature selection. However, instead of using an absolute threshold for SHAP values or a percentage of features, we propose to use a cumulative percentage threshold analogous to what can be done in Principal Component Analysis to achieve for example 95% of variance explained with a subset of principal components. The process of train-test splitting, imputation, model fitting, and evaluation was repeated with the new split-specific reduced feature set.

While 100 splits are useful to describe model performance to be expected on unseen data, fitting a final model on the entire set of patient records would maximize the use of information. The same methodology for feature reduction as previously in each individual split was performed after training a single model on the entire dataset. The final XGBoost model was retrained on the entire set of patient records with the reduced set of features. Since the identical methodology was used when creating the distributions of performance based on the test sets, the reported performance distributions are representative of the expected performance of the final model on unseen data.

### Reporting summary

Further information on research design is available in the Nature Research Reporting Summary linked to this article.

## Supplementary information

Reporting Summary

## Data Availability

The patient data that support the findings of this study are available from IBM Explorys but restrictions apply to the availability of these data, which were used under license for the current study, and so are not publicly available. The IBM Explorys database data are run by IBM who makes the data available for secondary use (e.g., for scientific research) on a commercial basis. Requests for access to the data should be sent to IBM Watson Health and not to the corresponding author.

## Code availability

Custom codes were made for the analysis using open source libraries (python 3.6.7, numpy 1.15.4, pandas 0.23.4, scikit-learn 0.20.1, scipy 1.1.0, shap 0.35.0, and xgboost 0.90). The custom codes are owned by IBM and cannot be shared for proprietary reasons. The predictive model is owned by IBM. However, IBM is willing to cooperate with researchers or research institutes on evaluation or further development of the algorithm and methods described in the publication, under the condition that the access to the model as well as sharing of output and results are clearly defined in a collaboration agreement.
