# Supplementary material for: Predicting critical state after COVID-19 diagnosis: model development using a large US electronic health record dataset
Source: NPJ Digit Med. 2021 Jul 20;4:113. doi: 10.1038/s41746-021-00482-9 (PMC8292360; doi:10.1038/s41746-021-00482-9)
Supplement: Supplementary file 1 — Reporting Summary [file 41746_2021_482_MOESM1_ESM.pdf]

nature research

Corresponding author(s): Yannick Klopfenstein

Last updated by author(s): Jun 17, 2021

Reporting Summary

Nature Research wishes to improve the reproducibility of the work that we publish. This form provides structure for consistency and transparency in reporting. For further information on Nature Research policies, see our Editorial Policies and the Editorial Policy Checklist.

Statistics

For all statistical analyses, confirm that the following items are present in the figure legend, table legend, main text, or Methods section.

n/a	Confirmed	
☐	☒	The exact sample size (*n*) for each experimental group/condition, given as a discrete number and unit of measurement
☐	☒	A statement on whether measurements were taken from distinct samples or whether the same sample was measured repeatedly
☒	☐	The statistical test(s) used AND whether they are one- or two-sided<br>*Only common tests should be described solely by name; describe more complex techniques in the Methods section.*
☐	☒	A description of all covariates tested
☐	☒	A description of any assumptions or corrections, such as tests of normality and adjustment for multiple comparisons
☐	☒	A full description of the statistical parameters including central tendency (e.g. means) or other basic estimates (e.g. regression coefficient) AND variation (e.g. standard deviation) or associated estimates of uncertainty (e.g. confidence intervals)
☒	☐	For null hypothesis testing, the test statistic (e.g. *F*, *t*, *r*) with confidence intervals, effect sizes, degrees of freedom and *P* value noted<br>*Give P values as exact values whenever suitable.*
☒	☐	For Bayesian analysis, information on the choice of priors and Markov chain Monte Carlo settings
☒	☐	For hierarchical and complex designs, identification of the appropriate level for tests and full reporting of outcomes
☒	☐	Estimates of effect sizes (e.g. Cohen's *d*, Pearson's *r*), indicating how they were calculated

Our web collection on statistics for biologists contains articles on many of the points above.

Software and code

Policy information about availability of computer code

Data collection	Our work was based on de-identified data from the Explorys database. The Explorys database is one of the largest clinical datasets in the world containing EHRs of clinical activity of around 64 million patients distributed across more than 360 hospitals in the US. This dataset contains data on patients in all 50 US states who seek care in healthcare systems which chose the IBM Enterprise Performance Management platform for their population and performance management and is not tied to particular insurers. Data were standardised and normalised using common ontologies, searchable through a Health Insurance Portability and Accountability Act (HIPAA)-enabled, de-identified dataset from IBM Explorys. Individuals were seen in multiple primary and secondary healthcare systems from 1999 to 2020 with a combination of data from clinical electronic medical records, health-care system outgoing bills, and adjudicated payer claims. The de-identified EHR data include patient demographics, diagnoses, procedures, prescribed drugs, vitals, and laboratory test results. Hundreds of billions of clinical, operational, and financial data elements are processed, mapped, and classified into common standards (e.g., ICD, SNOMED, LOINC, and RxNorm). As a condition of allowing the use of the de-identified data for research, these systems cannot be identified. The aggregated Explorys data were statistically de-identified to meet the requirements of 45 Code of Federal Regulations § 164.514(b), 1996 HIPAA, and 2009 Health Information Technology for Economic and Clinical Health (HITECH) standards. Business affiliation agreements were in place between all participating healthcare systems and Explorys regarding contribution of EHR data to the Explorys Platform and the use of these de-identified data. The Explorys dataset does not include data from patients who indicated at patient onboarding that they did not wish to have their data used for de-identified secondary use. Since the Explorys dataset consists of de-identified data for secondary use, the use of said dataset is not considered a human study and thus ethical approval was not required for the present work.
Data analysis	Custom codes were made for the analysis using open source libraries (python 3.6.7, numpy 1.15.4, pandas 0.23.4, scikit-learn 0.20.1, scipy 1.1.0, shap 0.35.0, and xgboost 0.90). The custom codes are owned by IBM and cannot be shared for proprietary reasons.

For manuscripts utilizing custom algorithms or software that are central to the research but not yet described in published literature, software must be made available to editors and reviewers. We strongly encourage code deposition in a community repository (e.g. GitHub). See the Nature Research guidelines for submitting code & software for further information.

Data

Policy information about availability of data

All manuscripts must include a data availability statement. This statement should provide the following information, where applicable:

- Accession codes, unique identifiers, or web links for publicly available datasets
- A list of figures that have associated raw data
- A description of any restrictions on data availability

The patient data that support the findings of this study are available from IBM Explorys but restrictions apply to the availability of these data, which were used under license for the current study, and so are not publicly available. The IBM Explorys database data are run by IBM who makes the data available for secondary use (e.g., for scientific research) on a commercial basis. Requests for access to the data should be sent to IBM Watson Health and not to the corresponding author.

Field-specific reporting

Please select the one below that is the best fit for your research. If you are not sure, read the appropriate sections before making your selection.

☒ Life sciences ☐ Behavioural & social sciences ☐ Ecological, evolutionary & environmental sciences

For a reference copy of the document with all sections, see nature.com/documents/nr-reporting-summary-flat.pdf

Life sciences study design

All studies must disclose on these points even when the disclosure is negative.

Sample size	The cohort included all patients in the Explorys database having a documented diagnosis of COVID-19 and a reported positive entry for a SARS-CoV-2 test, both since January 20, 2020.
Data exclusions	The full dataset was constructed based on COVID-19 diagnosis including binary prediction target labels for critical state and enriched by the various features. Patients with missing age or gender information were removed from the dataset.
Replication	To create a distribution and confidence intervals of the model performance, as performance may change depending on the choice of train-test split of the dataset for model creation, multiple non-random splits by time were created. The methodology of splitting by time is recommended in TRIPOD, as it allows for non-random variation between the train and test sets, since all records of the test data of each split come from a time window which has not been seen during training of the respective split. For each split, the dataset was split into a train set (80%) and a test set (20%). A sliding window was applied on the chronologically ordered patients to create 100 different splits, where the window of 20% width corresponded to the test set of the split. Thus, for the first split, the test set covered the chronologically first 20% of the data records (earliest cases), while the test set of the 100th split corresponded to the last 20% (most recent cases). The remaining data of a split (whether before or after the test set window) was used as a train set.
Randomization	Given the type of analysis and modeling approach, a non-random train-test split by time is preferable (according to TRIPOD) over a completely randomized train-test split.
Blinding	This work did not include any control groups, hence blinding is not applicable.

Reporting for specific materials, systems and methods

We require information from authors about some types of materials, experimental systems and methods used in many studies. Here, indicate whether each material, system or method listed is relevant to your study. If you are not sure if a list item applies to your research, read the appropriate section before selecting a response.

Materials & experimental systems

n/a	Involved in the study	
☒	☐	Antibodies
☒	☐	Eukaryotic cell lines
☒	☐	Palaeontology and archaeology
☒	☐	Animals and other organisms
☒	☐	Human research participants
☒	☐	Clinical data
☒	☐	Dual use research of concern

Methods

n/a	Involved in the study	
☒	☐	ChIP-seq
☒	☐	Flow cytometry
☒	☐	MRI-based neuroimaging
